# Bacterial genome sequencing in clinical microbiology: a pathogen-oriented review

**DOI:** 10.1007/s10096-017-3024-6

**Published:** 2017-06-21

**Authors:** F. Tagini, G. Greub

**Affiliations:** Institute of Microbiology, Department of Laboratory, University of Lausanne & University Hospital, Lausanne, Switzerland

**Keywords:** Genomics, Clinical microbiology, Next-generation sequencing, Whole-genome sequencing

## Abstract

In recent years, whole-genome sequencing (WGS) has been perceived as a technology with the potential to revolutionise clinical microbiology. Herein, we reviewed the literature on the use of WGS for the most commonly encountered pathogens in clinical microbiology laboratories: *Escherichia coli* and other Enterobacteriaceae, *Staphylococcus aureus* and coagulase-negative staphylococci, streptococci and enterococci, mycobacteria and *Chlamydia trachomatis*. For each pathogen group, we focused on five different aspects: the genome characteristics, the most common genomic approaches and the clinical uses of WGS for (i) typing and outbreak analysis, (ii) virulence investigation and (iii) in silico antimicrobial susceptibility testing. Of all the clinical usages, the most frequent and straightforward usage was to type bacteria and to trace outbreaks back. A next step toward standardisation was made thanks to the development of several new genome-wide multi-locus sequence typing systems based on WGS data. Although virulence characterisation could help in various particular clinical settings, it was done mainly to describe outbreak strains. An increasing number of studies compared genotypic to phenotypic antibiotic susceptibility testing, with mostly promising results. However, routine implementation will preferentially be done in the workflow of particular pathogens, such as mycobacteria, rather than as a broadly applicable generic tool. Overall, concrete uses of WGS in routine clinical microbiology or infection control laboratories were done, but the next big challenges will be the standardisation and validation of the procedures and bioinformatics pipelines in order to reach clinical standards.

## Introduction

Over the last decade, whole-genome sequencing (WGS) has been identified as one of the most promising techniques in clinical microbiology [1, 2]. Since the first bacterial genomes sequenced in 1995 [3, 4], it has come a long way and genome sequencing is now broadly implemented in research laboratories thanks to the rise of high-throughput sequencing [5]. Although its use in clinical microbiology increases, WGS is differentially implemented depending on the pathogen or the intended uses. Generally, clinical microbiology aims to provide a rapid detection and identification of a microorganism, for bacteria, combined or not with antimicrobial susceptibility testing (AST). Recent improvements of sequencing technologies with higher speed and output-to-cost ratios render WGS applicable for many aspects of clinical microbiology, including infectious disease control and epidemiology of pathogens [6, 7].

Even if WGS can be applied to all microorganisms (viruses, bacteria, parasites or fungi), this review focuses on clinical bacteriology. Very good review articles focusing on sequencing technologies or quality control have been published [5, 8, 9]. Herein, we aim to review the applications of WGS in clinical bacteriology focusing on the recent advances in terms of genomic approaches, applications for typing and outbreak, and in silico virulence-associated genes detection and antimicrobial susceptibility prediction for the most common pathogens encountered in blood cultures in our clinical microbiology laboratory [10], as well as for several intracellular bacteria of particular interest (Table 1). For antimicrobial susceptibility prediction based on genomic data, our review is aligned with the in-depth report of the European Committee on Antimicrobial Susceptibility Testing (EUCAST) by Ellington et al. that reviewed the literature on WGS prediction of phenotypic AST from genotypes [11]. We hope that our review will be useful for the clinical microbiologist wishing to obtain an update on the broad applications of WGS for very common pathogens.

**Table 1:** Items investigated in this review for each pathogen

Items	Pathogens
Genome characteristics	*Escherichia coli* and other Enterobacteriaceae
Genomic approach	*Staphylococcus aureus* and coagulase-negative staphylococci
Typing and outbreak	Streptococci and enterococci
Virulence	*Pseudomonas aeruginosa* and *Acinetobacter baumannii*
Antimicrobial susceptibility	*Mycobacterium tuberculosis* complex and other mycobacteria
	*Chlamydia trachomatis*

## *Escherichia coli* and other Enterobacteriaceae

### Genome characteristics

*Escherichia coli* is one of the most studied organisms in the world. Its genome size ranges from 4.6 Mb to 5.9 Mb for a median GC content of 50.6%, with 4200 to 5500 genes [12]. Overall, Enterobacteriaceae are characterised by a large variable genome with various intra-family horizontal gene transfer (HGT) or recombination, sometimes increased by the host’s medical conditions [13].

### Genomic approach

So far, WGS was applied mainly on extracted DNA from cultivated bacterial isolates. However, metagenomic shotgun amplification allowed the identification of foodborne pathogens directly from food samples [14–16]. Interestingly, Loman et al. used metagenomic shotgun amplification to investigate an outbreak of Shiga toxin-producing *E. coli*, but sensitivity remained low (67%) compared to cultures [17]. Hasman et al. performed WGS directly on clinical urine samples and successfully identified *E. coli*, and complete congruence with the regular microbiology work-up was observed [18].

### Typing and outbreak

*Escherichia coli* strains have been historically grouped into serotypes, biotypes, pathotypes and sequence types [12]. Serotypes (O and H antigens), pathotypes and sequence types [like multi-locus sequence typing (MLST) based on 7–8 housekeeping genes] can be inferred from WGS data [12, 19–21]. Moreover, WGS allows discrimination up to the single nucleotide polymorphisms (SNPs) level for real-time or retrospective investigation of outbreaks of *E. coli* [22–25], *Salmonella enterica* [26–31] or *Klebsiella* spp. [32–35]. Although variants detection allows the most sensitive discrimination between isolates based on DNA sequences, it is limited by the need for a reference genome or whole-genome alignment [36]. Moreover, they lack standardisation and usually do not allow straightforward comparison between studies [20]. New sequence typing methods, such as ribosomal MLST (rMLST, 53 loci) [37], core-genome MLST (cgMLST, >500 loci) or whole-genome MLST (wgMLST, all loci) have arisen since the era of WGS and allow typing up to the strain or clone levels [20]. The use of wgMLST was recently demonstrated by typing extended-spectrum beta-lactamase-producing Enterobacteriaceae [38]. These recent typing tools are available on EnteroBase (https://enterobase.warwick.ac.uk), an online database gathering metadata and genotypes inferred from genome assemblies for four gamma-proteobacteria (*Escherichia*/*Shigella*, *Salmonella*, *Yersinia* and *Moraxella*). Moreover, EnteroBase integrates a tool for *Salmonella* in silico serotyping developed by Yoshida et al. [39]. For *K. pneumoniae*, a cgMLST scheme was developed to type hypervirulent and multi-resistant strains [40]. Although there is controversy about differentiating the genus *Shigella* from *Escherichia* due to its genome similarities with enteroinvasive *E. coli* [41], a k-mer analysis coupled to MLST from inferred WGS data seems to be an effective discriminative approach [42].

### Virulence

Robins-Browne et al. raised the question of the relevance of pathotypes for intestinal pathogenic *E. coli* (IPEC) in the era of WGS [12]. Although pathotypes remain the subtyping system that is the most clinically relevant, WGS is able to: (i) predict pathotypes with accuracy (Table 2) and (ii) overcome the limitations of this classification, for instance with the emergence of strains with new pathogenic features, such as the enteroaggregative Shiga toxin-producing *E. coli* [12, 43]. Contrary to the obligate pathogen IPEC, extraintestinal pathogenic *E. coli* (ExPEC) are opportunistic pathogens and infections arise from the commensal microbiota. Therefore, an identification based on the presence/absence of virulence-associated genes in ExPEC genomes is not straightforward since host medical predispositions also play a major role in the pathogenesis, despite the description of many virulence-associated genes [44]. For *K. pneumoniae*, several plasmidic and chromosomal genes have been identified as virulence genes associated with community-acquired pyogenic liver abscesses [45, 46]. WGS can identify hypervirulent clones in a rapid manner, which can be of great use to prevent a clonal spread [40, 45, 47].

**Table 2:** Virulence-associated genetic determinants of the main *Escherichia coli* pathotypes

Gene/genomic region/plasmid	Functional role	Comments
LEE PAI	Genomic island containing *eae* (encoding gene for an adhesin) as well as effectors and structural proteins associated with a type III secretion system	EPEC defining region
pINV	Encodes for a type III secretion system and for effectors allowing intracellular survival	EIEC/*Shigella* defining plasmid
*est*, *elt*	Heat-stable (ST) and heat-labile (LT) enterotoxins	ETEC defining genes
*stx1*, *stx2*	Shiga toxins (verotoxins) 1 and 2	EHEC defining genes
*aggR*, *aatA*, *aaiC*	Transcriptional regulator, transporter protein and secreted protein	Associated with EAEC phenotype

Adapted from Robins-Browne et al. [12]

LEE PAI, Locus of enterocyte effacement pathogenicity island; EPEC, enteropathogenic *E. coli*; EIEC, enteroinvasive *E. coli*; ETEC, enterotoxigenic *E. coli*; EHEC, enterohaemorrhagic *E. coli*; EAEC, enteroaggregative *E. coli*

### Antimicrobial susceptibility

Overall, several studies reported more than 95% concordance between genotypic and phenotypic antimicrobial resistances for Enterobacteriaceae, such as *E. coli* and *K. pneumoniae* [48–50]. However, in a significant proportion of carbapenem-resistant *K. pneumoniae* and *E. cloacae* isolates, no carbapenemase could be detected, showing the presence of other resistance mechanisms [51]. Indeed, particular resistance mechanisms, such as modification in the membrane permeability or up-regulation of efflux pumps, will be harder to predict, and further studies are required to improve accuracy among heterogeneous datasets [11]. Furthermore, important limitations with short-read technologies remain for plasmid assemblies due to the inability of assemblers to deal with repeats [11]. They can be overcome using long-read sequencing to improve their detection [51–54] but the cost remains too high for most clinical laboratories. Finally, the particular case of *Salmonella* spp. needs to be further assessed due to the limited number of studies [11].

## *Staphylococcus aureus* and coagulase-negative staphylococci

### Genome characteristics

*Staphylococcus aureus* has a genome size that ranges from 2.6 to 3.1 Mb, with a median GC content of 32.8%. Coagulase-negative staphylococci (CoNS) have similar genome features to *S. aureus*. Mobile genetic elements represent 15–20% of the *S. aureus* genome, emphasising the important transfer of virulence factors and/or antimicrobial resistances that can happen between strains [55] or even between species [56–58].

### Genomic approach

The most common approach for *S. aureus* is WGS applied on extracted DNA from cultivated bacterial isolates. To our knowledge, no study reported culture-independent genome sequencing. Besides *S. aureus*, there are a limited number of studies on WGS application for CoNS in a clinical setting.

### Typing and outbreak

In terms of discriminatory power, WGS and SNP-based methods overcome all previous methods used for typing, such as pulsed-field gel electrophoresis (PFGE), 7-loci MLST and *spa* typing [59]. To ensure backward compatibility with traditional genotyping, *spa* types could be inferred from genome assemblies with 97% [60] and 99.1% [61] accuracy, although *spa* typing is based on the number and order of repeats, which can theoretically impair reliable genome assemblies from short reads. For SCC*mec*—a mobile genetic element carrying the methicillin resistance gene in *S. aureus* [62] that shows a great diversity and a high rate of recombination—typing can also be done using WGS and has the advantage to allow the detection of new types or subtypes, although multiplex polymerase chain reaction (PCR) and DNA microarray remain widely used [63]. During outbreak investigations, many studies could rule in or out a direct transmission of closely related isolates using SNP-based approaches [64–67]. As for Enterobacteriaceae, rMLST, cgMLST, wgMLST or even pan-genome MLST show high discriminatory power and, if used more often, could be of great use for standardisation and inter-study comparisons [20, 68, 69].

### Virulence

*Staphylococcus aureus* is a highly adapted pathogen and a number of its genes are related to virulence. WGS provides the possibility to screen the genomes for specific genes of interest, such as Panton–Valentine leucocidin (PVL) or superantigens encoding genes (Table 3), involved in severe clinical presentations, such as necrotising pneumonia or staphylococcal toxic shock syndrome [73]. Commercial multiplex PCRs or DNA microarrays are available and can already screen for some antibiotic resistance genes or particular virulence factors in a culture-independent manner. Their clinical utility remains controversial, although some authors recommend the adjunction of a clindamycin regimen for PVL^+^ necrotising pneumonia [73]. Thus, in the context of patient care, the use of WGS for virulence investigation remains limited if not done in a shorter time-to-result. Most of the CoNS virulence-associated genes known are genes related to biofilm or adherence to surface [74]. However, the pro-inflammatory and cytolytic phenol-soluble modulin (PSM) combined with the methicillin resistance island could play a critical role in CoNS sepsis pathogenesis [71].

**Table 3:** Main *Staphylococcus aureus* toxins encoded on the accessory genome

Gene/genomic region/plasmid	Functional role	Comments
PVL locus (*lukF-PV*, *lukS-PV*)	Pore-forming toxin targeting polymorphonuclear leucocytes	Phage-encoded toxin associated with necrotising pneumonia or severe skin and soft tissue formation
*lukD*/*E*, *lukG*/*H*	Pore-forming toxins targeting polymorphonuclear leucocytes	Located on a pathogenicity island (LukDE), they act synergistically with PVL
*psm-mec* locus	Cytolytic capacity, biofilm formation, methicillin resistance, cell spreading and expression of other virulence factors [70]	This locus may also be found in CoNS and could play a major role in CoNS sepsis [71]
*eta*, *etb*, *etd*	Exfoliative toxin A, B and D	Toxins involved in the pathogenesis of bullous impetigo and staphylococcal scaled-skin syndrome
*se(a-e)*, *se(g-j)*, *se(r-t)*, *sel(k-q)*, *sel(u-w)*, *tsst-1*	Staphylococcal enterotoxins and enterotoxin-like toxins	Superantigens associated with *S. aureus* food poisoning and toxic shock syndrome

Adapted from Grumann et al. [72]

### Antimicrobial susceptibility

Several studies report a high efficiency for in silico antimicrobial susceptibility testing [64, 75–78]. Mykrobe predictor, an online tool allowing a rapid discrimination between *S. aureus* and other staphylococci, predicts antimicrobial susceptibility with high sensitivity (99.1%) and specificity (99.6%) [79]. Moreover, the predictions are made from raw sequences and can be achieved in less than 3 min, thanks to a de Bruijn-based method. However, limitations for the antimicrobial susceptibility prediction remain (i) because of gaps in the knowledge and the important number of mechanisms of resistance existing for particular antibiotics such as aminoglycosides or glycopeptides [80, 81], as well as (ii) due to genetic instability with the loss of some mobile genetic elements such as *erm*(C) or the SCC*mec* cassette while passaging the isolate [11]. On the other hand, for mupirocin, mismatches between genotypic predictions and AST could be explained by laboratory variations. Indeed, those predicted resistant genotypes concerned isolates with a diameter of inhibition of 29 mm, whereas epidemiological cut-off (ECOFF) for the wild type is more than 30 mm for mupirocin. Therefore, it implies that the mupirocin zone diameter ECOFF needs to be revised [11]. For CoNS, studies comparing genotypic to phenotypic correlation remain limited.

## Streptococci and enterococci

### Genome characteristics

The median lengths are 1.8 Mb and 2.1 Mb for *Streptococcus pyogenes* and *S. pneumoniae*, respectively. Enterococci of medical importance, such as *Enterococcus faecalis* and *E. faecium*, have larger genomes, ranging from 2.6 to 3.4 Mb. The GC content for these two genera varies from 35% to 40%. Overall, streptococci and enterococci display high genome plasticity. HGT and homologous recombination can drive serotype modifications, as well as the spread of virulence factors and antibiotic resistance genes [82–84].

### Genomic approach

Regular WGS from bacterial culture is the standard. To our knowledge, no study reports a culture-independent WGS approach for streptococci detection. Hasman et al. could successfully identify *E. faecalis* by WGS directly from urine samples [18]. In addition, the *E. faecalis* complete genome sequence could be obtained directly by a metagenomic approach from stool samples by Morowitz et al. [85].

### Typing and outbreak

Molecular typing of *S. pyogenes* is classically done with the M-protein encoding gene (*emm*), as well as with the 7-loci MLST [86, 87]. However, for outbreak investigation, studies have shown the added value of WGS thanks to its high discriminatory power compared to other typing techniques [88–91]. *Streptococcus pneumoniae* serotypes are wildly used and important for epidemiological studies and vaccine development [92]. Interestingly, MLST is highly congruent with strain serotypes [93] and can be easily inferred from WGS data. Serotype prediction from WGS reads is possible thanks to PneumoCaT, a recently developed automated pipeline [94]. It holds the advantage of recognising particular cases of mixed serotypes or in the presence of new subtypes, possibly masked by regular methods. For enterococci, 7-loci MLST and SNP-based approaches are often used for epidemiological studies or outbreak investigations [95–101]. A cgMLST scheme for *E. faecium* was recently published by de Been et al. and reaches the same resolution as SNP-based approaches, which could facilitate standardisation and comparisons between laboratories [102].

### Virulence

Genomes of streptococci hold many genes related to virulence (Table 4) [103, 105]. However, in addition to the presence or absence of virulence-related genes, mutations in regulators, such as two-component systems, are often involved in increased virulence. Due to the complexity of the paths regulating virulence in streptococci, WGS data could benefit from being combined with RNA sequencing and in vivo study for outbreak investigations [89]. However, we hypothesise that having pipelines and databases of virulence-associated genes and mutations in regulators of virulence would be useful for public health surveillance or to prevent further complications of particular clinical presentations, for example by adding clindamycin to patients at risk of developing toxic shock syndrome for *S. pyogenes* based on the strain genotype.

**Table 4:** Main *Streptococcus pyogenes* virulence factors

Gene/genomic region/plasmid	Functional role	Comments
*hasA*, *hasB*, *hasC*	Hyaluronic acid capsule synthesis	Prevention of phagocytosis
*emm*	Antiphagocytic protein (M protein)	Sequence used for typing *S. pyogenes* isolates
*spyCEP*	Interleukin-8 protease	Inhibition of PMN leucocytes diapedesis
*sda1*	Streptodornase D (extracellular DNase)	Degradation of PMN DNA nets
*sagA*, *sagB*, *sagC*, *slo*	Streptolysin S and O	Lysis of red blood cells, epithelial cells, macrophages and PMN
*speA*, *speC*, *speH*, *speI*, *speJ*, *speL*/*M*, *ssa*, *SMEZ*	Superantigens	Involved in the pathogenesis of toxic shock syndrome or scarlet fever
*speB*	Cysteine protease	Tissue invasion and dissemination
*fbaA*, *sclA*	Adhesins	

Adapted from Cole et al. [103] and Reglinski and Sriskandan [104]

Many low-/non-virulent isolates hold virulence factors in their genome but their expression is under strong down-regulation. Mutations in two-component systems, such as *covR*/*S* or other regulators, have been associated with a dramatic up-regulation of most of those virulence-associated genes

PMN, Polymorphonuclear.

### Antimicrobial susceptibility

Many studies focus on antimicrobial resistance and rely to some extent on genomic data [11, 106, 107]. For instance, Howden et al. used WGS to investigate the transmission in hospitalised patients of vancomycin-resistant *E. faecium* (VREfm), which is, in fact, mainly driven by de novo generation and not only by nosocomial transmission as previously thought [108]. To extend the example of VRE, gene clusters involved in vancomycin resistance in enterococci such as *vanA* and *vanB* can be routinely screened using multiplex PCRs with a good correlation with phenotypic AST [109, 110]. By extension, WGS could be used to screen and detect all known *van* gene clusters. However, to our knowledge, no large studies compared WGS-based genotypic AST to phenotypic AST for streptococci or enterococci, despite the increasing knowledge on the genomic basis of antimicrobial resistances and the rise of multidrug-resistant streptococci and enterococci.

## *Pseudomonas aeruginosa* and *Acinetobacter baumannii*

### Genome characteristics

The *P. aeruginosa* genome size ranges from 6.1 to 7.5 Mb, with a median GC content of 66.2%. For *A. baumannii*, its genome size is shorter and varies from 3.7 to 4.3 Mb, with a median GC content of 39%. HGT and genome-wide homologous recombination plays a major role in these two successful and often multidrug-resistant opportunistic pathogens [111–114]. Plasmid-mediated antibiotic resistances play a major role in the transmission of antimicrobial resistances between isolates and species, which may be hard to assess based only on short reads sequencing, as discussed already for Enterobacteriaceae.

### Genomic approach

Most studies that investigated outbreaks used a regular culture-based approach for WGS. Nevertheless, culture-independent shotgun WGS was performed to investigate the composition of the microbiota of sputa sampled from patients with cystic fibrosis, without broad-range 16S rRNA PCR to avoid bias [115].

### Typing and outbreak

Recent studies showed the added value of WGS for outbreak investigation retrospectively or prospectively compared to other typing techniques for *P. aeruginosa* [116–121] and *A. baumannii* [122–126]. Thrane et al. made public a web tool (https://cge.cbs.dtu.dk/services/PAst-1.0/) for in silico determination of the *P. aeruginosa* serotype, which can be useful to detect or characterise outbreak clones [120]. A real-time WGS investigation of an outbreak in a neonatal intensive care unit was performed and could be used to trace back the index patient and the source of the outbreak [127]. Although it has not been used for *P. aeruginosa* so far, cgMLST was recently carried out for typing *A. baumannii* and successfully differentiated a clonal spread among other isolates [128].

### Virulence

WGS allowed indubitably a better understanding of acute or chronic *P. aeruginosa* and *A. baumannii* infections, and helps the development of new therapeutic approaches [129, 130]. However, besides its use for research or outbreak strain characterisation, a clinical application for the detection of virulence determinants to individualise treatments is currently too preliminary.

### Antimicrobial susceptibility

A large study comparing phenotypic and genotypic AST for *P. aeruginosa* reports 91% sensitivity and 94% specificity for both meropenem- and levofloxacin-resistant phenotypes prediction [131]. However, for amikacin, only 60% of non-susceptible isolates based on AST were congruent with the genomic findings. In contrast, Wright et al. observed high concordance with AST for predicted aminoglycoside and carbapenem susceptibility using 75 isolates of *A. baumannii* [132]. ARG-ANNOT (Antibiotic Resistance Gene-ANNOTation), a downloadable tool for the detection of antimicrobial resistances, was validated using 174 isolates of *A. baumannii* with 100% sensitivity and 100% specificity for the genes analysed, even when querying partial sequences [133]. Although good sensitivity/specificity may be reached based on the presence or absence of genes or point mutations in antibiotic target genes, major challenges remain in the prediction of chromosomal alterations, resulting in the modification of expression of genes, such as efflux pumps or intrinsic beta-lactamases [11]. More studies starting from strain collections remain to be done to compare phenotypic and genotypic methods for AST.

## *Mycobacterium tuberculosis* complex and other mycobacteria

### Genome characteristics

*Mycobacterium tuberculosis* complex (MTBC) has a clonal, monomorphic genome of approximately 4.3 to 4.4 Mb. HGT or recombination do not occur in MTBC, whereas it is an important driving force for evolution in other mycobacteria (*M. canetti* or non-tuberculosis mycobacteria, NTM) [134]. Thus, antimicrobial resistances can only occur from SNPs or insertion–deletion events in MTBC.

### Genomic approach

Although many genomic studies have been performed on classical mycobacterial culture, very concrete implementations were attempted in high-income countries [135, 136]. By performing WGS on positive MGIT, a complete report including species identification, in silico AST and calculation of genetic distance to detect outbreaks could be sent a median of 21 days faster than the final reference laboratory report [135]. Moreover, costs were 7% cheaper than the regular workflow for mycobacteria. Public Health England reports to be close to a broad implementation of WGS for the routine diagnosis of mycobacterial infections [137]. Finally, culture-independent WGS was performed directly on sputa. One study performed a proof of concept [138] and the other reported a high-quality sequencing for 20 out of 24 samples and highly concordant genotypic–phenotypic AST [139]. The time-to-AST was 14 days shorter than with other WGS workflows﻿﻿ using MGIT﻿﻿. In addition, two sequenced samples did not grow in regular culture, emphasising the added value of WGS performed directly on clinical samples [139].

### Typing and outbreak

Recent studies showed a higher resolution of WGS compared to other molecular typing techniques [140–143], such as restriction fragment length polymorphism (RFLP) [144], spoligotyping [145] or variable-number tandem repeats of mycobacterial interspersed repetitive units (MIRU-VNTR) [146]. Although, spoligotypes and MIRU-VNTR types can be determined from WGS, it is not a straightforward approach due to the repeats in the regions of interest, thus rendering assemblies difficult to make from short reads [147]. For public health, WGS was used to trace back outbreaks with high resolution, giving the possibility to identify clonal transmission between patients [148–150]. However, as discussed before, SNP-based approaches lack standardisation and inter-laboratory reproducibility. To tackle this issue, a cgMLST scheme was recently designed for MTBC [151].

### Virulence

Lessons from *M. tuberculosis* genomics allowed the identification of a large number of virulence genes, such as catalases, superoxide dismutase, as well as effectors of the type VII secretion system (ESAT-6, CFP10, recently renamed EsxA and EsxB) [152, 153]. However, the relevance to search for specific virulence genes is limited since MTBC populations are mainly clonal and assessment of virulence based on lineages holds more promise. There are seven lineages of MTBC of human health relevance [147]. Lineages 2 (particularly the modern Beijing sublineage) and 4 are the most widespread and are more virulent than lineages 1 and 6, with more severe clinical presentations, more transmissibility and less immunogenicity [154–157]. Given their restrictive geographic distribution, lineages 3, 5 and 7 are also likely to be less virulent [156]. Thus, knowing lineage informs on virulence and is of public health interest. In addition, automatic web tools can type and assign lineage to a strain from WGS raw data very quickly [158, 159].

### Antimicrobial susceptibility

Recent large studies compared AST with the detection of variants associated to antimicrobial resistances [160–162]. Moreover, several web-based automated tools, taking raw reads as input, are available [79, 158, 159, 163, 164]. Although sensitivity and specificity were high with the dataset used in these studies, the EUCAST study group identified several limitations [11]. (a) Low sensitivity for hetero-resistance is reported for molecular techniques [165] and coverage needs to be increased to overcome that, which, currently, would increase the cost and, thus, may not be suitable for a clinical microbiology laboratory setting. Moreover, most of the current pipelines are not designed to detect insertion–deletion events [166]. (b) Systematic errors may arise from poorly defined cut-offs for phenotypic AST that are used as standard for the validation of in silico AST. (c) Finally, genetic basis for antimicrobial resistance is not completely understood, particularly for non-essential genes involved in antimicrobial resistance, which means that WGS can mainly rule in rather than rule out antimicrobial resistance [11]. However, it is clear that WGS can improve the mycobacterial AST workflow and patient care by reducing dramatically the time to an effective antimicrobial regiment, despite it being unlikely that laboratories will dispense completely with phenotypic AST in the near future [11].

## *Chlamydia trachomatis*

### Genome characteristics

*Chlamydia trachomatis* has a small genome size, as a consequence of the adaptation to its intracellular habitat [167], of 1.0 Mb to 1.1 Mb, with a median GC content of 41.2%. Although there are evidences for HGT and especially for homologous recombination, these mechanisms seem to play a smaller role than point mutations for driving the evolution of *C. trachomatis* [168].

### Genomic approach

Culture-dependant approaches are time- and resource-consuming, due to the intracellular lifestyle of *C. trachomatis*. To tackle this issue, several studies successfully performed WGS directly on clinical samples by using various techniques: (i) immunomagnetic separation for targeted bacterial enrichment with multiple displacement amplification, (ii) capture RNA bait set, (iii) whole-genome amplification before WGS and (iv) multiplexed microdroplet PCR enrichment technology [169–172]. A limitation for the clinical use of the first technique could be the lysis buffer, which is present in some commercial devices, and may prevent the binding of antibodies to the major outer membrane protein (MOMP) [173].

### Typing and outbreak

*Chlamydia trachomatis* was historically classified by MOMP-based serology. Serovars are clinically important because they determine the tissue tropism of the infection (serovars A–C, ocular; D–K, urogenital and ocular; L1–L3, lymph nodes) [168]. In recent years, PCR of the *ompA*, the gene encoding for the MOMP, was developed for typing but exhibited very low epidemiological resolution [174]. The multi-locus variable-number tandem repeat (VNTR) analysis (MLVA) system and various MLST schemes as well as the multi-locus typing DNA array were developed, which provide more reliable topologies [175–177]. WGS was shown to have a higher resolution than regular phylogenies based on MLST [178].

### Virulence

Numerous genes and variants were associated with specific tissue tropism or pathogenic effect [168]. However, besides a straightforward use of WGS to build robust core-genome phylogenies and to infer serovar from *ompA* to predict tropism, there is currently not enough knowledge on specific virulence factors that could have a clinical value.

### Antimicrobial susceptibility

Although treatment failures have been reported, they are not likely due to antimicrobial resistance, which will hopefully remain rare [179]. Thus, there is currently a limited need for in silico antimicrobial resistance predictions for *C. trachomatis*.

## Discussion

**Fig. 1:**
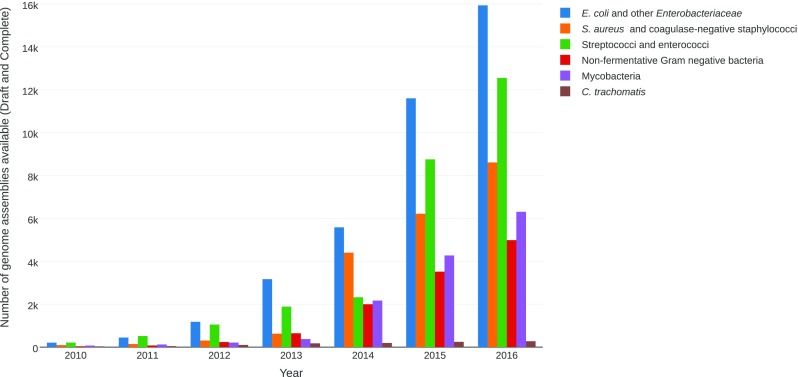
Number of genome assemblies available in the National Center for Biotechnology Information (NCBI) database per year

For all the major pathogens investigated during this review, we can observe an increasing number of publically available genomes (Fig. 1). Along with this trend, our review shows the development of various WGS-based approaches, as well as attempts of their implementation in a clinical microbiology routine. Knowledge on the genomics of the pathogens is a prerequisite before any clinical use and important features need to be kept in mind for each microorganism. Although horizontal gene transfer or recombination events are very frequent in most pathogens, they do not occur in *M. tuberculosis*. This is critical because HGT and recombination have a large impact on the transmission of virulence factors, antimicrobial resistance genes and on serovar modifications. Concerning the genomic approaches, WGS is regularly performed on cultivated isolates, but an increasing number of studies report culture-independent WGS, which could speed up the clinical laboratory workflow, particularly to decrease the time to genotypic AST. A straightforward and broadly recognised use of WGS is for the investigation of outbreaks and is nowadays broadly implemented in clinical microbiology and infection control laboratories. Although SNP-based methods have shown great successes, new typing approaches such as rMLST or cgMLST schemes, which offer standardisation and comparability between laboratories, are available for an increasing number of organisms. Moreover, they were shown to be highly reproducible and accurate [180]. Mellmann et al. used cgMLST to monitor prospectively the transmission of methicillin-resistant *S. aureus*, VRE, multidrug-resistant *E. coli* and multidrug-resistant *P. aeruginosa*. This approach was efficient and cost-effective in the setting of a majority of multi-bed rooms and because of the possibility to reduce a systematic isolation recommended by German guidelines [181]. Diseases pathogenesis is extremely diverse and complex. For most pathogens, there is no straightforward approach to predict an isolate’s virulence based on its genotype. Indeed, host factors as well as modification of the expression of virulence-associated genes add another layer of complexity. However, WGS can provide a map of the virulome, which can sometimes be determining for a patient’s care, for instance, by precisely determining the *E. coli* pathotype. The EUCAST subcommittee reports that there is currently not enough evidence to support clinical decision-making based on genotypic AST [11]. However, for mycobacteria, WGS implementation for diagnosis, in silico AST and outbreak investigation was shown to be successful and cost-effective, with a rapid turnaround time, saving weeks or even months of cultures [135].

Finally, for an implementation in clinical microbiology, WGS-based methods will need standardised and validated (i) procedures, (ii) quality control and (iii) subsequent bioinformatics pipelines. Moreover, they will need to be in line with the clinical requirements for data protection.
